# Management and prediction of immune-related adverse events for PD1/PDL-1 immunotherapy in colorectal cancer

**DOI:** 10.3389/fphar.2023.1167670

**Published:** 2023-04-28

**Authors:** Liting Sun, Cong Meng, Xiao Zhang, Jiale Gao, Pengyu Wei, Jie Zhang, Zhongtao Zhang

**Affiliations:** ^1^ Department of General Surgery, Beijing Friendship Hospital, Capital Medical University and National Clinical Research Center for Digestive Diseases, Beijing, China; ^2^ Department of Radiology, Beijing Friendship Hospital, Capital Medical University, Beijing, China

**Keywords:** colorectal cancer, immunotherapy, PD-1/PD-L1 inhibitors, immune-related adverse events, prediction

## Abstract

Programmed cell death protein (PD-1) is an important immunosuppressive molecule, which can inhibit interaction between PD-1 and its ligand PD-L1, further enhancing the T cell response and anti-tumor activity, which is called immune checkpoint blockade. Immunotherapy, represented by immune checkpoint inhibitors, has opened up a new era of tumor treatment and is gradually being applied to colorectal cancer recently. Immunotherapy was reported could achieve a high objective response rate (ORR) for colorectal cancer with high microsatellite instability (MSI), thus opening up a new era of colorectal cancer immunotherapy. Along with the increasing use of PD1 drugs in colorectal cancer, we should pay more attention to the adverse effects of these immune drugs while seeing the hope. Immune-related adverse events (irAEs) caused by immune activation and immune homeostasis during anti-PD-1/PD-L1 therapy can affect multi-organ and even be fatal in serious cases. Therefore, understanding irAEs is essential for their early detection and appropriate management. In this article, we review the irAEs that occur during the treatment of colorectal cancer patients with PD-1/PD-L1 drugs, analyze the current controversies and challenges, and point out future directions that should be explored, including exploring efficacy predictive markers and optimizing the paradigm of individualized immunotherapy.

## 1 Introduction

Colorectal cancer is the third most common malignancy worldwide (Zheng et al., 2022). The latest global statistics show that in 2020 there were 1,148,515 new cases of colon cancer and 732,210 new cases of rectal cancer (Wei et al., 2020; Zheng et al., 2022). For decades, surgery, radiotherapy, and chemotherapy have been the main weapons used by physicians to fight colorectal cancer. However, there are problems with current treatment, especially for some patients who are not candidates for surgery in the advanced stage. In recent years, immunotherapy has emerged and the advent of immune checkpoint inhibitors (ICI) has opened up a new era in oncology treatment.

In recent years, many findings have confirmed that immunosuppressive molecules such as cytotoxic T lymphocyte-associated antigen 4 (CTLA4), PD-1and its ligand PD-L1 are seen to be significantly overexpressed in the immune microenvironment of tumor patients (Mellman et al., 2011). PD-1 is an important immunosuppressive molecule. It regulates the immune system and promotes self-tolerance by down-regulating the immune system response to human cells, as well as by suppressing T-cell inflammatory activity. Significant upregulation of expression is seen in certain tumors, where PD-1 binding to its receptor PD-L1 initiates programmed death of T cells, allowing tumor cells to acquire immune escape (Postow et al., 2018; Seidel et al., 2018). By inhibiting the interaction between PD-1 and PD-L1, T-cell responses are enhanced and thus anti-tumor activity is increased, i.e., immune checkpoint blockade (Larkin et al., 2015). Checkpoint inhibitors targeting the PD-1 pathway are now approved for the treatment of a variety of tumors (Larkin et al., 2015; Motzer et al., 2015).

In 2015, Dung T.'s team first used pd1 drugs for the treatment of patients with dMMR/MSI-H metastatic colorectal cancer, and 10 previously treated patients were treated with the PD-1 inhibitor pembrolizumab, and the results showed an ORR of 40%, indicating that this group of patients may benefit from this treatment (Le et al., 2015). Since then, we have seen a new hope for the treatment of colorectal cancer, and subsequently, more teams have conducted related studies, all of which resulted in good therapeutic outcomes, further demonstrating the efficacy of PD-1 drugs in mCRC patients with dMMR (Overman et al., 2017; André et al., 2020; Stein et al., 2021; Haag et al., 2022). Besides, immunologic drugs in CRC may have good efficacy in patients with locally advanced rectal cancer (LARC). Currently, the first-line treatment for patients with LARC is still surgery combined with radiotherapy, which involves R0 survival and anal preservation. The VOLTAGE study investigated neoadjuvant immunotherapy with Nivolumab after long-course simultaneous radiotherapy for locally advanced rectal cancer, and the pathologic complete response (pCR) rate in the dMMR group reached 60% (Bando et al., 2022). Similar results have been obtained from some other studies in China and abroad that patients with locally progressive colorectal cancer receiving neoadjuvant immunotherapy can achieve a high pCR rate (Shamseddine et al., 2020; Lin et al., 2021; Hu et al., 2022). It can be seen that immunotherapy will undoubtedly play a great power in the future for both mCRC patients and LARC patients, and the application of PD-1 drugs in colorectal cancer is incomparably bright.

Along with the increasing use of PD-1 drugs in colorectal cancer, we must be concerned about the adverse effects of this class of immune drugs while seeing hope. By unbalancing the immune system, immune checkpoint blockade favors the development of autoimmune manifestations, also known as irAEs. Most of these adverse events can be managed by steroids to counteract lymphocyte activation. However, although steroid use causes irAEs to subside, the associated immunosuppression may impair the antitumor response (Kuehn et al., 2014; Darnell et al., 2020). It has been reported in the literature that severe irAEs not only do not benefit patients but may lead to death. In addition, the onset of irAEs is difficult to predict and can occur even after treatment is discontinued and persist for a long time. The expected frequency of AEs in immunotherapy, chemotherapy, and other treatment modalities differs due to the unique mechanism of action of ICIs. Therefore, understanding irAEs is crucial for their early detection and appropriate management and is more likely to further guide the use of PD-1 drugs in the field of colorectal cancer.

We conducted a systematic literature search of the pubMed, MEDLINE, Cochrane Library, EMBASE, China National Knowledge Infrastructure (CNKI), Wanfang Database, VIP Medical Information System, and China Biomedical Database (CBM) from inception to 15 December 2022. The search terms were composed of the following medical themes (MeSH) and additional conditions: (colorectal cancer/colorectal neoplasms/colorectal tumor) AND (programmed cell death protein/PD-1/PD-L1) AND (immune-related adverse events/irAEs). Furthermore, manual studies would be conducted to find potential references. Language was not an obstacle to publication.

## 2 Clinical application and management of irAEs in CRC patients treated with ICI

The 2021 version of the NCCN Guidelines changes the previous recommendations for detecting MMR/MSI status. The guidelines recommend universal MMR or MSI testing for all patients with a personal history of colon or rectal cancer. In addition to serving as a predictive marker for immunotherapy in advanced CRC settings, MSI/MMR status can also help identify individuals with Lynch syndrome and inform adjuvant treatment decisions in patients with stage II CRC. Firstly, we summarized the different applications of PD-1/PD-L1 inhibitors in a clinical study of CRC patients (Table 1).

**TABLE 1:** Summary of PD-1/PD-L1 inhibitors in clinical studies in colorectal cancer patients. mCRC, Metastatic colorectal cancer; LARC, locally advanced rectal cancer

Author	Year	Trial population	Drug	N	AE Grade1-2	AE Grade3-4
Le et al. (2015)	2015	mCRC	Pembrolizumab	41	23%	17%
Overman et al. (2017)	2017	mCRC	Nivolumab	74	49%	20%
André et al. (2020)	2020	mCRC	Pembrolizumab	149	41%	56%
Stein et al. (2021)	2021	mCRC	Avelumab	43	NA	NA
Haag et al. (2022)	2022	mCRC	Pembrolizumab	20	NA	5%
Bando et al. (2022)	2021	mCRC	Nivolumab	38	NA	NA
Brahmer et al. (2010)	2010	mCRC	MDX-1106	14	36%	0
O'Neil et al. (2017)	2017	mCRC	Pembrolizumab	23	35%	4%
Ott et al. (2017)	2017	mCRC	Pembrolizumab	24	64%	16%
Morris et al. (2017)	2017	mCRC	Nivolumab	37	NA	14%
Shamseddine et al. (2020)	2020	LARC	Avelumab	13	NA	23%
Lin et al. (2021)	2021	LARC	Camrelizumab	27	97%	27%
Hu et al. (2022)	2022	LARC	Toripalimab	34	67%	9%
Wang et al. (2022a)	2022	LARC	Toripalimab	130	NA	NA
Cercek et al. (2022)	2022	LARC	Dostarlimab	12	75%	0

### 2.1 PD-1/PD-L1 inhibitors in metastatic CRC

In the initial phase I study of MDX-1106 (anti-PD-1 antibody), irAEs were specifically concerned (Brahmer et al., 2010). In this study, 14 metastatic CRC patients were well tolerated to the maximum planned dose of 10 mg/kg. Among the 14 patients, no grade≥3 irAEs occurred. However, gastrointestinal toxicities attributed to MDX-1106 were observed. Out of 39 patients including CRC, one experienced grade 3 ascites, and one experienced grade 3 colitis. Two other patients experienced grade 2 stomatitis. None of the patients received treatment for these gastrointestinal toxicities (Brahmer et al., 2010).

The KEYNOTE-016 study reported in 2015, in which 41 patients with metastatic colorectal cancer were given treatment with pembrolizumab 10 mg/kg every 14 days, showed 40 cases (98%) of adverse events and 17 cases (41%) of grade III or higher. Special adverse reactions included thyroiditis or hypothyroidism (10%), asymptomatic pancreatitis (15%), diarrhea (24%), intestinal obstruction (7%), and upper respiratory tract infection (7%) (Le et al., 2015).

In a cohort of 20 PD-L1 positive advanced CRC patients, the irAEs of pembrolizumab treatment were systematically analyzed (O'Neil et al., 2017). The most important category of irAEs is pneumonitis including interstitial lung disease and acute interstitial pneumonitis. Pembrolizumab treatment was suggested to be held if any pneumonitis events reached grade 2 and pembrolizumab treatment was permanently discontinued if any pneumonitis events were above grade 3 (O'Neil et al., 2017). While a similar course of action was applied to hepatitis (O'Neil et al., 2017). When grade 3 colitis, rash, uveitis, iritis, endocrine AEs, thyroid disorders, neurological AEs, or hematological AEs occurred, pembrolizumab treatment was held (O'Neil et al., 2017). Among the 23 advanced colorectal carcinoma patients treated with pembrolizumab, one patient experienced grade 4 increased blood bilirubin and pembrolizumab was discontinued as suggested (O'Neil et al., 2017).

In an open-label, multicenter, phase 2 study of Nivolumab in microsatellite instability-high/mismatch repair-deficient (MSI-H/dMMR) colorectal cancer patients (CheckMate 142), 98.6% of patients were reported with all-cause AEs. Grade 3 or 4 AEs were reported in 20.3% of patients and five (6.8%) patients discontinued treatment due to AEs. Of note, one patient who received a steroid taper for grade 3 colitis still died 10 days after their last dose (Overman et al., 2017). In the 4-year follow-up from CheckMate 142, Grade 3 or 4 AEs were reported to increase from 20.3% to 32% and AEs that lead to discontinuation increased from 6.8% to 13% (Overman et al., 2017). 5 patients discontinued treatment due to drug-related adverse events, including ALT elevation, colitis, duodenal ulcer, acute kidney injury, and stomatitis (n = 1 each) (Overman et al., 2017).

In the KEYNOTE-177 study, 153 MSI-H CRC patients in the trial group were given 200 mg of pembrolizumab every 3 weeks, and the study reported adverse reactions in 149 (97%) patients in the trial group. Common adverse reactions included diarrhea, fatigue, nausea, loss of appetite, and alopecia in 22% of grade 3 and higher adverse reactions, and immune-related adverse reactions included hypothyroidism, colitis, hyperthyroidism, pneumonia, and adrenal insufficiency in 9% of grade 3 and higher immune-related adverse reactions (André et al., 2020).

In the PICCASSO study, 20 patients with refractory colorectal cancer were treated with pembrolizumab and maraviroc (8 cycles) followed by pembrolizumab monotherapy. The study results reported that the most common adverse reactions during treatment in 20 patients were fatigue (30%), rash and pruritus (15%), and elevated AST (10%). Only one patient had a grade 3 adverse reaction, manifesting as hyperglycemia; one other patient had hypothyroidism and one patient had keratitis (Haag et al., 2022).

In a trial of pembrolizumab for a patient with recurrent carcinoma of the anal canal, four out of 24 patients developed grade 3 adverse events and continued therapy after symptomatic treatment (Ott et al., 2017). In a clinical trial of Nivolumab for a patient with metastatic anal cancer, five out of 37 patients experienced grade 3 adverse events. One patient developed grade 2 pneumonitis and subsequently received steroid therapy and a temporary treatment break while another patient received a short course of corticosteroids for the treatment of nivolumab-related autoimmune hypothyroidism (Morris et al., 2017).

### 2.2 PD-1/PD-L1 inhibitors in locally advanced rectal cancer

Neoadjuvant therapy for CRC is mainly aimed at locally advanced rectal cancer and some resectable metastatic CRC. Traditional neoadjuvant therapies include chemotherapy, radiotherapy, targeted therapy, and combination therapy. At present, neoadjuvant therapy for CRC is mainly radiotherapy, combined with chemotherapy drugs, and the addition of PD-1 to neoadjuvant therapy for cancer is a new attempt. In a prospective single-arm multicenter phase II trial by Shamseddine’s team, mFOLFOX6 plus avelumab (10 mg/kg) was given every 2 weeks for a further 6 cycles to 13 patients with progressive colorectal cancer who had undergone 5 cycles of total 25 Gy radiotherapy included in the study. A total of 27 adverse reactions were recorded in 13 patients, with the most common adverse reactions being diarrhea and fatigue (36%). Three grade 3 adverse events, one small bowel obstruction, one *Salmonella* colitis, and one acute kidney injury (Shamseddine et al., 2020).

In a prospective, single-arm phase II trial by Lin’s team in 2021, 30 patients with locally progressive rectal adenocarcinoma were given a 5 × 5 Gy dose of radiotherapy and two 21-day treatments of CAPOX in combination with camrelizumab1 week after the start of radiotherapy, followed by radical surgery. The study results reported that the most common treatment-related adverse reactions were leukopenia (80%), and reactive cutaneous capillary endothelial hyperplasia (73%). Immune-related adverse reactions were all grade 1–2, the most common being reactive cutaneous capillary endothelial hyperplasia in 22 of 27 patients (81%); hypothyroidism was seen in two other patients (Lin et al., 2021).

In a single-center phase II study conducted in China, the participants received Toripalimab 3 mg/kg intravenously on day 1, with or without celecoxib 200 mg orally twice daily from day 1–14 of each 14-day cycle, for six cycles before surgical resection. 26 (76%) of 34 patients had at least one treatment-related adverse event during the study. The most common grade 1–2 treatment-related adverse events were hyperthyroidism (18%), fatigue (12%), increase in aspartate aminotransferase levels (12%), abdominal pain (12%), and pruritus (2%) in the combination group; and fatigue (24%), pruritus (18%), nausea (18%), and rash (18%) in the Toripalimab monotherapy group (Hu et al., 2022).

TORCH is a randomized, prospective, multicentre, double-arm, phase II trial of short-course radiotherapy (SCRT) combined with chemotherapy and immunotherapy in LARC. The consolidation arm will receive SCRT, followed by 6 cycles of capecitabine plus oxaliplatin (CAPOX) and Toripalimab. The induction arm will receive 2 cycles of CAPOX and Toripalimab, then receive SCRT, followed by 4 cycles of CAPOX and Toripalimab. Among 130 patients, the grade 3–4 immune-related toxicities were 7.7% (Wang et al., 2022a).

In a phase II study with published results in 2022, a total of 12 patients have completed treatment with dostarlimab and have undergone at least 6 months of follow-up. Adverse events of any grade occurred in 12 of the 16 patients (75%; 95% CI, 48–92). No adverse events of grade 3 or higher were reported. The most common adverse events of grade 1 or 2 included rash or dermatitis (in 31% of the patients), pruritus (in 25%), fatigue (in 25%), and nausea (in 19%). Thyroid-function abnormalities occurred in 1 patient (6%) (Cercek et al., 2022).

### 2.3 Management of irAEs in CRC patients

Due to the broad range of irAEs in CRC patients treated with ICI, the management of irAEs is drawing increasing attention (Darnell et al., 2020). Immune-related toxicities vary in onset, severity, and potential biology, and they may affect a wide range of organs, thus requiring specialized management approaches (Brahmer et al., 2018). Among the various irAEs, skin toxicity such as rash, pruritus and vitiligo are generally the most common and earliest to occur. Although most dermal toxicities are transient, their higher incidence is associated with patient quality of life. Gastrointestinal toxicity is also one of the most common complications. The most common clinical manifestations of immune-associated gastrointestinal toxicity range from very frequent and/or loose stools to symptoms of colitis (e.g., stool mucus, abdominal pain, fever, rectal bleeding). Compared to the first two symptoms, immunotherapy-associated pneumonia is a less frequent but potentially serious toxic adverse reaction. Moreover, immune-related endocrine adverse events occasionally occur, usually in the form of symptoms or abnormal laboratory parameters. In addition, there are some diseases with lower morbidity, including cardiovascular system, neurological system, renal system, etc. They can occur at any time during a patient’s treatment, most commonly during the first 3 months of therapy. Management of irAEs is primarily focused on glucocorticoid therapy. Most symptomatic irAEs (except for endocrine disease) are treated well with several weeks of glucocorticoid therapy. In addition, although most irAEs regress, some become chronic and may require lifelong treatment such as hormone supplementation or immunosuppression (Conroy and Naidoo, 2022).

There are few relevant clinical studies, and the methods of treatment and management are mainly proposed and summarized by experienced specialists. The need for clinical management is primarily determined by the severity of the organs and irAEs involved, and management includes discontinuation of ICI therapy and initiation of topical, oral, or parenteral steroids. Steroid-related medications are currently commonly used for treatment, but the jury is still out on the optimal initial steroid dose and duration of steroid therapy, with the expectation that more prospective evidence will support this in the future. In addition, there are some expert recommendations for relatively severe irAEs, and perhaps with the use of immunosuppressive drugs.

According to the 2022 updated ESMO guidelines, irAEs management generally consists of four sequential steps: i) diagnosis and grading of irAEs, ii) ruling out differential diagnoses and pre-immunosuppression work-up, iii) selecting the appropriate immunosuppression strategy for grade 2 events and iv) active evaluation at 72 h to adapt treatment (Haanen et al., 2022). The recommendations in the guide mainly include IR-skin toxicity, IR-endocrinopathies, IR-hepatotoxicity, IR-cholangitis, IR-pancreatic toxicity, IR-gastrointestinal toxicity, IR-pulmonary toxicities, IR-rheumatological toxicity, IR-neurological toxicity, IR-cardiovascular toxicities, IR-renal toxicity, IR-major hematological toxicity and IR-ocular toxicity (Haanen et al., 2022).

Apart from the common ICI-induced irAEs, some rare, but severe and fatal, irAEs were observed in CRC patients treated with ICI. Here, we summarized rare irAEs according to clinical management in CRC patients treated with ICI. Severe necrotizing myositis was observed in CRC patients treated with nivolumab plus ipilimumab combination therapy. After discontinuation of ICI treatment, intravenous methylprednisolone combined with intravenous immunoglobulins was provided and most of the symptoms were resolved (Tauber et al., 2019). Nivolumab plus regorafenib treatment in a CRC patient resulted in immune-related keratitis (Su et al., 2022). Glucocorticoids and autologous serum were used as a diagnostic treatment and the patient recovered from irAEs after one-month treatment (Su et al., 2022). A patient with metastatic adenocarcinoma of the colon receiving atezolizumab developed acute macular neuro retinopathy, the symptom resolved after 5 weeks of oral steroids but atezolizumab treatment was discontinued and the patient died 5 months after the onset of visual symptoms (Emens et al., 2019). Atezolizumab plus cobimetinib treatment resulted in a high incidence of treatment discontinuation for CRC patients than atezolizumab monotherapy (21% V.S. 4%) in colorectal cancer patients due to irAEs (Eng et al., 2019). Recently, a CRC patient who received tislelizumab experienced a cooccurrence of severe myasthenia gravis, myocarditis, and rhabdomyolysis (Wang et al., 2022b). Methylprednisolone and intravenous immunoglobulin therapy were applied and the patient responded well (Wang et al., 2022b).

Endocrine irAEs did not require corticosteroid therapy according to the guidelines (Panhaleux et al., 2022). However, hormone therapy facilities the recovery of endocrine disorders developed in CRC patients during ICI treatment. It has been reported that pembrolizumab caused adrenocorticotropic hormone deficiency in a cecal mucinous cancer patient and cortisol treatment was promptly effective (Bekki et al., 2020). Primary adrenal insufficiency was observed in a patient treated with nivolumab and hydrocortisone effectively corrected the hyponatremia (Deligiorgi and Trafalis, 2020). Diabetes mellitus was observed in a CRC patient treated with pembrolizumab and insulin therapy and management of electrolytes were provided (Kichloo et al., 2020). Ipilimumab and nivolumab treatment caused anterior hypophysitis in a CRC patient and stress dose IV hydrocortisone levothyroxine attenuated the symptoms. The patient was rechallenged with nivolumab monotherapy and remains asymptomatic (Jing et al., 2020).

## 3 Prediction of irAEs in CRC patients treated with ICI

The common adverse reactions of the antibody class of PD-1 and PD-L1 drugs currently in common use can be manifested in the skin, endocrine, gastrointestinal, and cardiac organs. Generally speaking, adverse reactions usually appear 2–3 months after drug administration, and the first manifestation is mostly seen in the skin. In summary, some common immunotherapy-related adverse reactions include fatigue, rash, colitis, hyper/hypothyroidism, anemia, decreased neutrophils, and elevated amylase. Some specific complications of immunotherapy are also of concern, including neurological, allergic, pneumonia, renal, and ocular adverse reactions, which can have very serious effects when they happen. Hence, the prediction of irAEs as well as patient monitoring would provide favorable results for patients who experienced irAEs and needed a rechallenge. Current guidelines on adverse reactions to immunotherapy focus on the identification of adverse reactions and corresponding treatment regimens, and it would certainly be more beneficial for patients to be able to predict this outcome in advance. According to the existing research, there are two main types of prediction methods, multi-omics analysis, and serological biomarkers, respectively.

### 3.1 Multi-omics analysis

**FIGURE 1:**
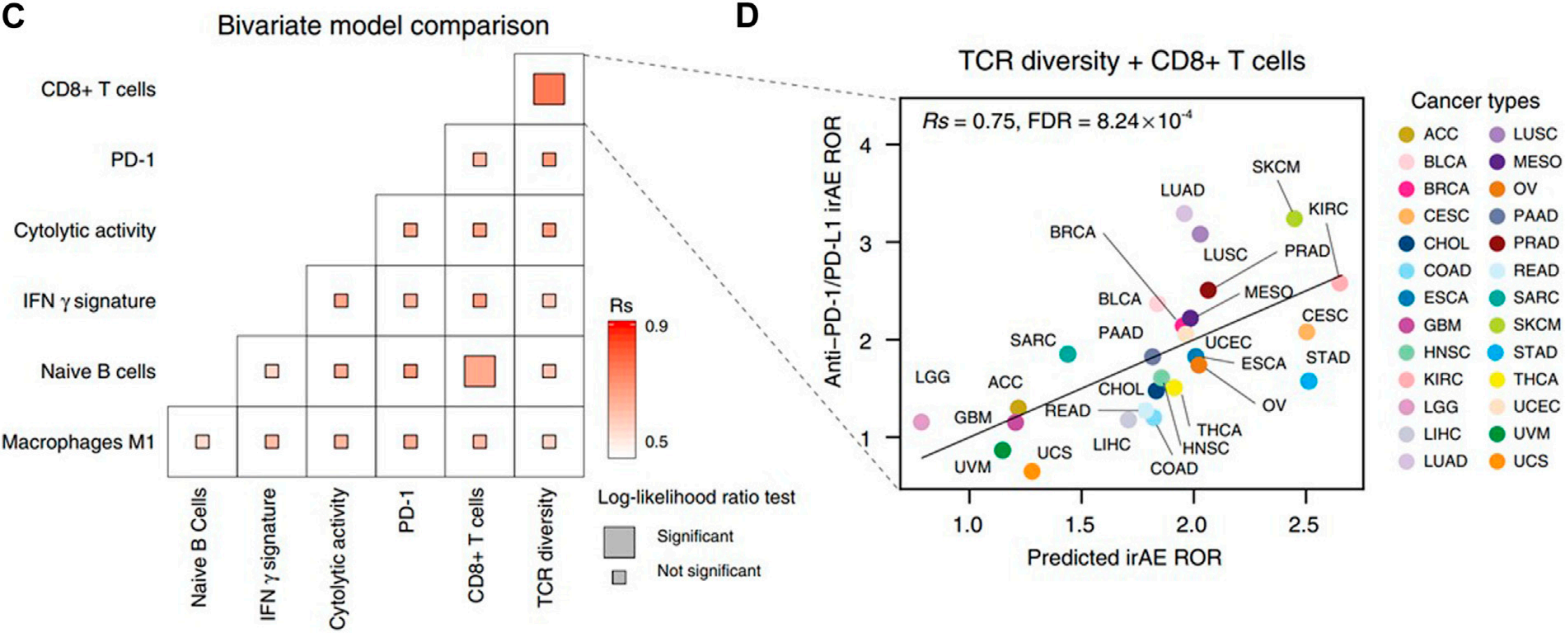
**(C)** Comparison of performance of bivariate models in predicting irAEs for all combinations of the top ten irAEs ROR significantly correlated genes. Spearman correlation (Rs) was calculated between the predicted and observed irAEs ROR. The shade of the square indicates the Rs, and the size indicates the significance of the log-likelihood ratio test. **(D)** Combined effect of LCP1 and ADPGK bivariate model (Spearman correlation, Rs = 0.91, FDR = 7.94 × 10^−9^). The equation of the bivariate regression model is 0.37× LCP1 + 0.70× ADPGK—9.10. The image quoted from Nat Commun., Multi-omics prediction of immune-related adverse events during checkpoint immunotherapy, Jing Y et al., 2020 October 2; 11(1):4946 (Jing et al., 2020).

The initial analysis of predicative biomarkers for irAEs in CRC patients is a multi-omics prediction method that analyzed mRNA, miRNA, lncRNA, and protein expression and non-silent gene mutations across 26 cancer types including rectum adenocarcinoma and colon adenocarcinoma (Jing et al., 2020). Researchers sought to identify additional predictive factors for irAEs by conducting a comprehensive screening across mRNA, miRNA, lncRNA and protein expression, and non-silent gene mutations across 26 cancer types. The results show that the lymphocyte cytosolic protein 1 (LCP1), which is involved in T-cell activation, achieved the highest correlation coefficient (Rs = 0.82, FDR = 6.69 × 10^−3^, Figure 1). In the study, the authors finally came up with a bivariate regression model of LCP1 and ADPGK expression in tumor tissues that can accurately predict irAEs. This was followed by a retrospective study of cancer patients receiving anti-PD-1/PD L1 therapy at Beijing Shijitan Hospital, which culminated in a preliminary validation of the model’s accuracy in the real world (Jing et al., 2020).

**FIGURE 2:**
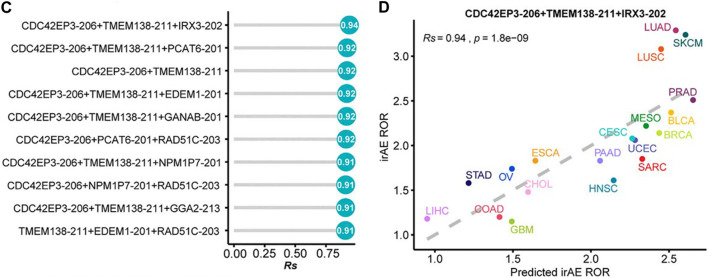
**(C)** Comparison of performance of bivariate and trivariate models in predicting irAEs for all combinations of the top ten irAEs ROR significantly correlated splicing isoforms. Rs was calculated between predicted and observed irAEs ROR. **(D)** Combination of CDC42EP3-206, TMEM138-211, and IRX3-202 to predict irAE risk. The dot color represents the cancer type. The dashed line represents the linear fit. The image quoted from Front Pharmacol., Pan-Cancer Analysis Reveals Alternative Splicing Characteristics Associated With Immune-Related Adverse Events Elicited by Checkpoint Immunotherapy, He X et al., 2021 November 24; 12:797852 (He et al., 2021).

A pan-cancer transcriptomic analysis showed that expression levels of splicing factors were predictive of irAEs risk (He et al., 2021). The researchers detected and characterized the relationship between the expression of splicing isoforms and irAE ROR using pancancer data. The top ten irAE ROR significantly correlated splicing isoforms were utilized for building the irAE ROR predictions. Combinations between any two or three of these predictors were then evaluated by Spearman correlation and goodness of fit using the log-likelihood ratio test. Notably, the combination of CDC42EP3-206 and TMEM138-211 with most of the other predictors achieved better predictive performance (Figure 2) (He et al., 2021).

In addition, another study used a similar approach in another comprehensive analysis of cellular and molecular factors in 9,104 patients with 21 types of cancer. Researchers identified 11 new predictors of irAEs by screening global multi-omics data. Among them, IRF4 showed the highest correlation and the best predictive performance of the IRF4-TCL1A-SHC-pY317 trivariate model (Zhang et al., 2022). The genome-wide association study was also utilized to identify single nucleotide polymorphisms that are associated with the risk of irAEs (Udagawa et al., 2022).

Recently, a genome-wide association study of 1,751 patients on ICI across 12 cancer types was performed and rs16906115 near IL7 was found and replicated in three independent studies (Groha et al., 2022). Mechanically, the authors showed that patients carrying the IL7 germline variant exhibited significantly increased lymphocyte stability after ICI initiation, which was itself predictive of downstream irAEs (Groha et al., 2022).

### 3.2 Serological biomarkers

Serological biomarkers have long been explored to predict the incidence of irAEs due to their cheap and easy availability compared to expensive histological tests. Adam et al. found that absolute lymphocyte count was correlated with the risk of irAEs in colon cancer patients treated with nivolumab or pembrolizumab (Diehl et al., 2017). Their data suggest that patients with higher baseline lymphocyte counts have a greater risk for irAEs, whereas patients with lymphopenia at baseline and persistent lymphopenia while on therapy have a shorter time to progression on these agents (Diehl et al., 2017). The results of a study also demonstrate that peripheral blood inflammatory markers can serve as predictors of treatment response and prognosis in patients with advanced GC and CRC receiving anti–PD-1 therapy (Fan et al., 2021). It has been shown that the rate of irAEs is higher in CRC patients with low platelet-to-lymphocyte ratio patients (Fan et al., 2021).

In pan-cancer studies including colon cancer patients showed that a lower relative lymphocyte count, higher albumin level, and higher absolute eosinophil count were significantly associated with the occurrence of irAEs (Bai et al., 2021). Importantly, the study showed that a higher lactate dehydrogenase level was an independent predictor of irAEs of grade ≥3 (Bai et al., 2021). However, a larger validation cohort is desperately needed to verify the efficacy of these biomarkers in colorectal cancer.

**FIGURE 3:**
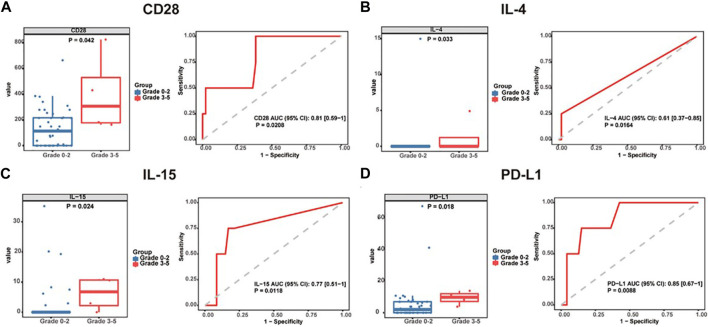
Baseline serum cytokine levels are significantly associated with irAE development and severity. Box plots (left) showing the distribution of serum cytokines **(A)** CD28, **(B)** IL-4, **(C)** IL-15, and **(D)** PD-L1 in grade 0–2 and 3-5 patients. ROC curve (right) analysis of sensitivity and specificity of serum cytokines **(A)** CD28, **(B)** IL-4, **(C)** IL-15, and **(D)** PD-L1 from baseline, to distinguish between grade 0–2 and 3-5 irAEs. The median of each group and *p*-value were calculated using the Mann-Whitney U test (*p* < 0.05). irAEs: immune-related adverse events, ROC: receiver operating characteristics. The image quoted from Front Immunol., Serological biomarkers predict immune-related adverse events and clinical benefit in patients with advanced gastrointestinal cancers, Wang Y et al., 2022 September 8; 13:987568 (Wang et al., 2022c).

In a gastrointestinal cancer cohort, serum CD28, IL-4, IL-15, and PD-L1 were significantly elevated in patients with grade 3–5 irAEs (Wang et al., 2022c). Interestingly, serum IL-6 was found higher in patients with thyroiditis and colitis. IL-22 and stem cell factor (SCF) levels were found higher in patients with colitis. IL-1a, IL-21, LIF, and PIGF-1 levels were significantly higher in patients with myositis and BTLA, GM-CSF, IL-4, PD-1, PD-L1, and TIM-3 levels were significantly higher in patients with rash (Figure 3) (Wang et al., 2022c). Since it is of special significance to predict organ-specific irAEs, this work provided a breakthrough point to make a personalized prediction of irAEs.

There is previous evidence of a relationship between gut microbiota composition and response to treatment in patients with irAEs. The use of fecal microbiota transplantation for the treatment of colitis has also been explored and has been successfully used to treat immunotherapy-associated colitis in a series of cases17 (Wang et al., 2018). In the future, models that use gastrointestinal flora in conjunction with relevant biomarker information to predict irAEs may also be further explored.

## 4 Summarize

With the widespread use of PD-1 and PD-L1 drugs in various oncology areas, there is a growing body of data on the safety and efficacy studies of these drugs. Immune-related adverse events (irAEs) during anti-PD-1 or PD-L1 antibody therapy are caused by disturbances in immune activation and immune homeostasis, can affect any organ system, and in some cases can be fatal. Pneumonia is the most common fatal irAEs, with a mortality rate of 10% and accounting for 35% of anti-PD-1/PD-L1 treatment-related deaths. Myocarditis is the most fatal irAEs, with a 50% mortality rate. Therefore, predictive biomarkers of irAEs are needed to determine the benefit-risk ratio for patients receiving anti-PD-1/PD-L1 therapy. Several relevant basic studies have been performed to investigate potential predictors of irAEs risk in patients receiving anti-PD-1/PD-L1 therapy in 26 tumor types by integrating real-world pharmacovigilance and molecular-omics data. It may provide the oncology field with a way to identify potential biomarkers of irAEs in cancer immunotherapy. In the future, we look forward to more large-scale clinical data to validate the utility of these methods in the field of colorectal cancer so that we can intervene early in high-risk groups for targeted surveillance and timely individualized and balanced treatment.

## References

[B1] André T. Shiu K. K. Kim T. W. Jensen B. V. Jensen L. H. Punt C. (2020). Pembrolizumab in microsatellite-instability-high advanced colorectal cancer. N. Engl. J. Med. 383 (23), 2207–2218. 10.1056/nejmoa2017699 33264544

[B2] Bai R. Chen N. Chen X. Li L. Song W. Li W. (2021). Analysis of characteristics and predictive factors of immune checkpoint inhibitor-related adverse events. Cancer Biol. Med. 18 (4), 1118–1133. Epub ahead of print. PMID: 34259422; PMCID: PMC8610160. 10.20892/j.issn.2095-3941.2021.0052 34259422 PMC8610160

[B3] Bando H. Tsukada Y. Inamori K. Togashi Y. Koyama S. Kotani D. (2022). Preoperative chemoradiotherapy plus nivolumab before surgery in patients with microsatellite stable and microsatellite instability-high locally advanced rectal cancer. Clin. Cancer Res. 28 (6), 1136–1146. PMID: 35063964; PMCID: PMC9365382. 10.1158/1078-0432.CCR-21-3213 35063964 PMC9365382

[B4] Bekki T. Takakura Y. Kochi M. Konemori Y. Oki K. Yoneda M. (2020). A case of isolated adrenocorticotropic hormone deficiency caused by pembrolizumab. Case Rep. Oncol. 13 (1), 200–206. PMID: 32308578; PMCID: PMC7154275. 10.1159/000505687 32308578 PMC7154275

[B5] Brahmer J. R. Drake C. G. Wollner I. Powderly J. D. Picus J. Sharfman W. H. (2010). Phase I study of single-agent anti-programmed death-1 (MDX-1106) in refractory solid tumors: Safety, clinical activity, pharmacodynamics, and immunologic correlates. J. Clin. Oncol. 28 (19), 3167–3175. Epub 2010 Jun 1. PMID: 20516446; PMCID: PMC4834717. 10.1200/JCO.2009.26.7609 20516446 PMC4834717

[B6] Brahmer J. R. Lacchetti C. Schneider B. J. Atkins M. B. Brassil K. J. Caterino J. M. (2018). Management of immune-related adverse events in patients treated with immune checkpoint inhibitor therapy: American society of clinical oncology clinical Practice guideline. J. Clin. Oncol. 36 (17), 1714–1768. Epub 2018 Feb 14. PMID: 29442540; PMCID: PMC6481621. 10.1200/JCO.2017.77.6385 29442540 PMC6481621

[B7] Cercek A. Lumish M. Sinopoli J. Weiss J. Shia J. Lamendola-Essel M. (2022). PD-1 blockade in mismatch repair-deficient, locally advanced rectal cancer. N. Engl. J. Med. 386 (25), 2363–2376. Jun 5. PMID: 35660797; PMCID: PMC9492301. 10.1056/nejmoa2201445 35660797 PMC9492301

[B8] Conroy M. Naidoo J. (2022). Immune-related adverse events and the balancing act of immunotherapy. Nat. Commun. 13 (1), 392. PMID: 35046403; PMCID: PMC8770784. 10.1038/s41467-022-27960-2 35046403 PMC8770784

[B9] Darnell E. P. Mooradian M. J. Baruch E. N. Yilmaz M. Reynolds K. L. (2020). Immune-related adverse events (irAEs): Diagnosis, management, and clinical pearls. Curr. Oncol. Rep. 22 (4), 39. 10.1007/s11912-020-0897-9 32200442

[B10] Deligiorgi M. V. Trafalis D. T. (2020). Reversible primary adrenal insufficiency related to anti-programmed cell-death 1 protein active immunotherapy: Insight into an unforeseen outcome of a rare immune-related adverse event. Int. Immunopharmacol. 89, 107050. Epub 2020 Oct 15. PMID: 33069924. 10.1016/j.intimp.2020.107050 33069924

[B11] Diehl A. Yarchoan M. Hopkins A. Jaffee E. Grossman S. A. (2017). Relationships between lymphocyte counts and treatment-related toxicities and clinical responses in patients with solid tumors treated with PD-1 checkpoint inhibitors. Oncotarget 8 (69), 114268–114280. PMID: 29371985; PMCID: PMC5768402. 10.18632/oncotarget.23217 29371985 PMC5768402

[B12] Emens L. A. Davis S. L. Oliver S. C. N. Lieu C. H. Reddy A. Solomon S. (2019). Association of cancer immunotherapy with acute macular neuroretinopathy and diffuse retinal venulitis. JAMA Ophthalmol. 137 (1), 96–100. PMID: 30383154; PMCID: PMC6439799. 10.1001/jamaophthalmol.2018.5191 30383154 PMC6439799

[B13] Eng C. Kim T. W. Bendell J. Argilés G. Tebbutt N. C. Di Bartolomeo M. Atezolizumab with or without cobimetinib versus regorafenib in previously treated metastatic colorectal cancer (IMblaze370): A multicentre, open-label, phase 3, randomised, controlled trial. Lancet Oncol. 2019;20(6):849–861. 10.1016/S1470-2045(19)30027-0 Epub 2019 Apr 16 Erratum in: Lancet Oncol 2019 Jun;20(6):e293 PMID: 31003911. 31003911

[B14] Fan X. Wang D. Zhang W. Liu J. Liu C. Li Q. (2021). Inflammatory markers predict survival in patients with advanced gastric and colorectal cancers receiving anti-PD-1 therapy. Front. Cell Dev. Biol. 9, 638312. PMID: 33791296; PMCID: PMC8005614. 10.3389/fcell.2021.638312 33791296 PMC8005614

[B15] Groha S. Alaiwi S. A. Xu W. Naranbhai V. Nassar A. H. Bakouny Z. (2022). Germline variants associated with toxicity to immune checkpoint blockade. Nat. Med. 28 (12), 2584–2591. Epub 2022 Dec 16. PMID: 36526723. 10.1038/s41591-022-02094-6 36526723 PMC10958775

[B16] Haag G. M. Springfeld C. Grün B. Apostolidis L. Zschäbitz S. Dietrich M. (2022). Pembrolizumab and maraviroc in refractory mismatch repair proficient/microsatellite-stable metastatic colorectal cancer - the PICCASSO phase I trial. Eur. J. Cancer 167, 112–122. Epub 2022 Apr 12. PMID: 35427833. 10.1016/j.ejca.2022.03.017 35427833

[B17] Haanen J. Obeid M. Spain L. Carbonnel F. Wang Y. Robert C. (2022). Management of toxicities from immunotherapy: ESMO clinical Practice guideline for diagnosis, treatment and follow-up. Ann. Oncol. 33 (12), 1217–1238. Epub 2022 Oct 18. PMID: 36270461. 10.1016/j.annonc.2022.10.001 36270461

[B18] He X. Yu J. Shi H. (2021). Pan-cancer analysis Reveals alternative splicing characteristics associated with immune-related adverse events elicited by checkpoint immunotherapy. Front. Pharmacol. 12, 797852. PMID: 34899357; PMCID: PMC8652050. 10.3389/fphar.2021.797852 34899357 PMC8652050

[B19] Hu H. Kang L. Zhang J. Wu Z. Wang H. Huang M. (2022). neoadjuvant PD-1 blockade with toripalimab, with or without celecoxib, in mismatch repair-deficient or microsatellite instability-high, locally advanced, colorectal cancer (PICC): A single-centre, parallel-group, non-comparative, randomised, phase 2 trial. Lancet Gastroenterol. Hepatol. 7 (1), 38–48. Epub 2021 Oct 22. PMID: 34688374. 10.1016/S2468-1253(21)00348-4 34688374

[B20] Jing Y. Liu J. Ye Y. Pan L. Deng H. Wang Y. (2020). Multi-omics prediction of immune-related adverse events during checkpoint immunotherapy. Nat. Commun. 11 (1), 4946. PMID: 33009409; PMCID: PMC75. 10.1038/s41467-020-18742-9 33009409 PMC7532211

[B21] Kichloo A. Albosta M. S. McMahon S. Movsesian K. Wani F. Jamal S. M. (2020). Pembrolizumab-induced diabetes mellitus presenting as diabetic ketoacidosis in a patient with metastatic colonic adenocarcinoma. J. Investig. Med. High. Impact Case Rep. 8, 2324709620951339. PMID: 32830561; PMCID: PMC7448133. 10.1177/2324709620951339 PMC7448133 32830561

[B22] Kuehn H. S. Ouyang W. Lo B. Deenick E. K. Niemela J. E. Avery D. T. (2014). Immune dysregulation in human subjects with heterozygous germline mutations in CTLA4. Science 345, 1623–1627. 10.1126/science.1255904 25213377 PMC4371526

[B23] Larkin J. Hodi F. S. Wolchok J. D. (2015). Combined nivolumab and ipilimumab or monotherapy in untreated melanoma. N. Engl. J. Med. 373 (13), 1270–1271. 10.1056/NEJMc1509660 26398076

[B24] Le D. T. Uram J. N. Wang H. Bartlett B. R. Kemberling H. Eyring A. D. (2015). PD-1 blockade in tumors with mismatch-repair deficiency. N. Engl. J. Med. 372 (26), 2509–2520. PMID: 26028255; PMCID: PMC4481136. 10.1056/NEJMoa1500596 26028255 PMC4481136

[B25] Lin Z. Cai M. Zhang P. Li G. Liu T. Li X. (2021). Phase II, single-arm trial of preoperative short-course radiotherapy followed by chemotherapy and camrelizumab in locally advanced rectal cancer. J. Immunother. Cancer 9 (11), e003554. Erratum in: J Immunother Cancer. 2022 Feb;10(2): PMID: 34725214; PMCID: PMC8562535. 10.1136/jitc-2021-003554 34725214 PMC8562535

[B26] Mellman I. Coukos G. Dranoff G. (2011). Cancer immunotherapy comes of age. Nature 480 (7378), 480–489. PMCID: PMC3967235. 10.1038/nature10673 22193102 PMC3967235

[B27] Morris V. K. Salem M. E. Nimeiri H. Iqbal S. Singh P. Ciombor K. (2017). Nivolumab for previously treated unresectable metastatic anal cancer (NCI9673): A multicentre, single-arm, phase 2 study. Lancet Oncol. 18 (4), 446–453. Epub 2017 Feb 18. PMID: 28223062; PMCID: PMC5809128. 10.1016/S1470-2045(17)30104-3 28223062 PMC5809128

[B28] Motzer R. J. Escudier B. McDermott D. F. George S. Hammers H. J. Srinivas S. (2015). Nivolumab versus everolimus in advanced renal-cell carcinoma. N. Engl. J. Med. 373, 1803–1813. 10.1056/NEJMoa1510665 26406148 PMC5719487

[B29] O'Neil B. H. Wallmark J. M. Lorente D. Elez E. Raimbourg J. Gomez-Roca C. (2017). Safety and antitumor activity of the anti-PD-1 antibody pembrolizumab in patients with advanced colorectal carcinoma. PLoS One 12 (12), e0189848. PMID: 29284010; PMCID: PMC5746232. 10.1371/journal.pone.0189848 29284010 PMC5746232

[B30] Ott P. A. Piha-Paul S. A. Munster P. Pishvaian M. J. van Brummelen E. M. J. Cohen R. B. (2017). Safety and antitumor activity of the anti-PD-1 antibody pembrolizumab in patients with recurrent carcinoma of the anal canal. Ann. Oncol. 28 (5), 1036–1041. PMID: 28453692; PMCID: PMC5406758. 10.1093/annonc/mdx029 28453692 PMC5406758

[B31] Overman M. J. McDermott R. Leach J. L. Lonardi S. Lenz H. J. Morse M. A. (2017). nivolumab in patients with metastatic DNA mismatch repair-deficient or microsatellite instability-high colorectal cancer (CheckMate 142): An open-label, multicentre, phase 2 study. Lancet Oncol. 18 (9), 1182–1191. Epub 2017 Jul 19. Erratum in: Lancet Oncol. 2017 Sep;18(9):e510. PMID: 28734759; PMCID: PMC6207072. 10.1016/S1470-2045(17)30422-9 28734759 PMC6207072

[B32] Panhaleux M. Espitia O. Terrier B. Manson G. Maria A. Humbert S. (2022). Anti-programmed death ligand 1 immunotherapies in cancer patients with pre-existing systemic sclerosis: A postmarketed phase IV safety assessment study. Eur. J. Cancer 160, 134–139. Epub 2021 Nov 19. PMID: 34810048. 10.1016/j.ejca.2021.10.018 34810048

[B33] Postow M. A. Sidlow R. Hellmann M. D. (2018). Immune-related adverse events associated with immune checkpoint blockade. N. Engl. J. Med. 378 (2), 158–168. 10.1056/NEJMra1703481 29320654

[B34] Seidel J. A. Otsuka A. Kabashima K. (2018). Anti-PD-1 and anti-CTLA-4 therapies in cancer: Mechanisms of action, efficacy, and limitations. Front. Oncol. 8, 86. 10.3389/fonc.2018.00086 29644214 PMC5883082

[B35] Shamseddine A. Zeidan Y. H. El Husseini Z. Kreidieh M. Al Darazi M. Turfa R. (2020). Efficacy and safety-in analysis of short-course radiation followed by mFOLFOX-6 plus avelumab for locally advanced rectal adenocarcinoma. Radiat. Oncol. 15 (1), 233. PMID: 33028346; PMCID: PMC7542723. 10.1186/s13014-020-01673-6 33028346 PMC7542723

[B36] Stein A. Simnica D. Schultheiß C. Scholz R. Tintelnot J. Gökkurt E. (2021). PD-L1 targeting and subclonal immune escape mediated by PD-L1 mutations in metastatic colorectal cancer. J. Immunother. Cancer 9 (7), e002844. PMID: 34315821; PMCID: PMC8317124. 10.1136/jitc-2021-002844 34315821 PMC8317124

[B37] Su Y. Li G. Xu J. Zheng J. Jiao J. Zhang J. (2022). Immune-related keratitis is a rare complication associated with nivolumab treatment in a patient with advanced colorectal cancer: A case report. Front. Oncol. 12, 1021713. PMID: 36457511; PMCID: PMC9706189. 10.3389/fonc.2022.1021713 36457511 PMC9706189

[B38] Tauber M. Cohen R. Laly P. Josselin L. André T. Mekinian A. (2019). Severe necrotizing myositis associated with long term anti-neoplastic efficacy following nivolumab plus ipilimumab combination therapy. Clin. Rheumatol. 38 (2), 601–602. Epub 2018 Nov 19. PMID: 30456528. 10.1007/s10067-018-4373-y 30456528

[B39] Udagawa C. Nakano M. H. Yoshida T. Ohe Y. Kato K. Mushiroda T. (2022). Association between genetic variants and the risk of nivolumab-induced immune-related adverse events. Pharmacogenomics 23 (16), 887–901. Epub 2022 Oct 21. PMID: 36268685. 10.2217/pgs-2022-0113 36268685

[B40] Wang S. Peng D. Zhu H. Min W. Xue M. Wu R. (2022). Acetylcholine receptor binding antibody-associated myasthenia gravis, myocarditis, and rhabdomyolysis induced by tislelizumab in a patient with colon cancer: A case report and literature review. Front. Oncol. 12, 1053370. PMID: 36568231; PMCID: PMC9773380. 10.3389/fonc.2022.1053370 36568231 PMC9773380

[B41] Wang Y. Shen L. Wan J. Zhang H. Wu R. Wang J. (2022). Short-course radiotherapy combined with CAPOX and toripalimab for the total neoadjuvant therapy of locally advanced rectal cancer: A randomized, prospective, multicentre, double-arm, phase II trial (TORCH). BMC Cancer 22 (1), 274. PMCID: PMC8922781. 10.1186/s12885-022-09348-z 35291966 PMC8922781

[B42] Wang Y. Wiesnoski D. H. Helmink B. A. Gopalakrishnan V. Choi K. DuPont H. L. Fecal microbiota transplantation for refractory immune checkpoint inhibitor-associated colitis. Nat. Med. 2018;24(12):1804–1808. Epub 2018 Nov 12. Erratum in: Nat Med. 2018 Nov 27;: PMID: 30420754; PMCID: PMC6322556. 10.1038/s41591-018-0238-9 30420754 PMC6322556

[B43] Wang Y. Zou J. Li Y. Jiao X. Wang Y. Zhuo N. (2022). Serological biomarkers predict immune-related adverse events and clinical benefit in patients with advanced gastrointestinal cancers. Front. Immunol. 13, 987568. PMID: 36159840; PMCID: PMC9492966. 10.3389/fimmu.2022.987568 36159840 PMC9492966

[B44] Wei W. Zeng H. Zheng R. Zhang S. An L. Chen R. (2020). Cancer registration in China and its role in cancer prevention and control. Lancet Oncol. 21 (7), e342–e349. 10.1016/s1470-2045(20)30073-5 32615118

[B45] Zhang L. Shi Y. Han X. (2022). Immunogenomic correlates of immune-related adverse events for anti-programmed cell death 1 therapy. Front. Immunol. 13, 1032221. PMID: 36505471; PMCID: PMC9733471. 10.3389/fimmu.2022.1032221 36505471 PMC9733471

[B46] Zheng R. Zhang S. Zeng H. Wang S. Sun K. Chen R. (2022). Cancer incidence and mortality in China, 2016. J. Natl. Cancer Cent. 2 (1), 1–9. 10.1016/j.jncc.2022.02.002 PMC11256658 39035212

